# The Etiology of Tetanus

**Published:** 1891-08

**Authors:** 


### The Etiology of Tetanus.

In the last number of the *Annales de l'Institut Pasteur* there appears (from the Bacteriological Laboratory of Val-de-Grace) a most interesting paper on tetanus by Drs. Vaillard and Vincent, which appears to throw very considerable light on the subject of tetanus, and to clear up a number of points and observations that have hitherto been enshrouded in obscurity. After describing the organism, and identifying it with that already made familiar through the papers of recent writers, the authors give it as their firm opinion that in cases of artificial inoculation of pure cultures it is always the poison introduced along with the bacillus, and not the organism itself, that acts upon the animal. This, indeed, seems to be probable, as they are able to prove that almost inconceivably minute doses of this poison, which they compare with snake poison, are quite sufficient to produce all the symptoms of most acute tetanus; in fact, it was almost impossible, from some of the cultures that they obtained, to administer a dose that was not lethal.

An exceedingly interesting feature brought out in the course of their work is that in no case was the poison developed as soon as the organism began to grow; in fact, gelatine cultures of the tetanus bacillus were never capable of producing toxic symptoms until liquefaction of the gelatine had commenced, when spores were demonstrated to have been formed, and when the peculiar disagreeable odor so characteristic of tetanus cultures had become perceptible. They associate both the odor and the peptonizing power with the formation of the poison in the cultures. That it was due merely to the presence of the spores that the material was poisonous they demonstrated by heating their cultures to a temperature of 62° C. for a short time (a temperature which is quite incapable of interfering with the vitality of the spores), when it was found that cultures so heated and introduced by inoculation into a rabbit or guinea-pig failed to produce any tetanus, thus proving that although the spores are not killed the poison has been destroyed by the heat. The spores were proved to be living by making fresh cultures from them in artificial media; after a time they grew luxuriantly, and if left to grow eight or ten days produced another crop of the poison. By simply washing away the poison from the spores with distilled water they also obtained similar results, for, although the spores could still develop and form the specific poison in artificial media, they were, when inoculated, incapable of giving rise to any symptoms of tetanus. From the reaction to heat of a substance they were able to separate, and from its resemblance to the diastases in other respects, they concluded that they have obtained from tetanus cultures the true tetanus poison, a poison, however, that can not be formed by the tetanus bacillus in healthy tissues. The micro-organisms are here so rapidly attacked by the leucocytes that they are rendered *hors de combat* before they have time to form their poison.

It has long been known that the tetanus bacillus could not develop in the tissues except, apparently, in the presence of other organisms, and the suggestion is offered that these other organisms act in one of two ways; they either paralyze the activity of the leucocytes, or they draw off, as it were, their attention and activity from the tetanus bacillus, thus allowing it sufficient time to develop its characteristic products. It is interesting to note that Drs. Vaillard and Vincent consider that in many respects the tetanus bacillus is extremely like the diphtheria bacillus, the method of action on and in the organism being essentially the same in the two cases, the above factors, in all probability, playing a part in diphtheria much as in the case of tetanus; and it is evident that in studying the one poison much light may be thrown on the other. Behring and Katasato appreciated this fact, and combined their forces to work out the question of immunity in these two diseases. It is obvious, however, from a consideration of some of the points that are indicated in this paper, that there are many sources of fallacy that will have to be eliminated before the ultimate explanation of the condition of immunity in protected animals can be given. The facts that this poison is active in such extraordinarily minute quantities, and that micro-organisms are able to grow with such difficulty in the human tissues, allow us to hope that extremely minute changes in the blood may be quite sufficient to secure the alteration or breaking-down of the virulent poison even when it has become diffused throughout the system. So long as the organism is localized to the wound there is, of course, more chance of coping successfully with the disease although here, as in other diseases, there always appears to be a possibility of the poison exerting such a paralyzing influence on the cells that usually take up foreign substances, that secondary septic conditions may be liable to occur even when the action of the tetanic poison can be antagonized so far as its primary effects on the cells are concerned. One question appears to be set at rest, and that is, as regards tetanus and diphtheria the ptomaines have had their day whatever may become of the products of other organisms. It may be accepted that here, at any rate, we have some subtle poison which although it has not yet been actually separated, has become so far isolated that it may be taken as proved that it is not an alkaloid or basic poison. A most remarkable feature is, that in peptonizing gelatine with the filtrate from a meat-broth culture of the tetanus bacillus, the poisonous properties are lost to a certain degree in direct proportion to the amount of gelatine that is peptonized; this, taken in conjunction with the fact that the properties are not developed until the gelatine begins to liquefy, has led Drs. Vaillard and Vincent to suppose that the same agent that peptonizes the gelatine is the active agent in bringing about the development of the toxic symptoms of tetanus.—*The Lancet.*

---

"PURE science is the knowledge of principles and deductions. When this knowledge is reduced to a set of precepts, with practical skill as the basis, it becomes an art. When knowledge is made the foundation for practical results to be secured, it becomes applied science. A man may study physiology or pathology as abstract sciences, but when he makes a practical application of that knowledge in the curing of disease, he is engaged in professional work. The practice of dentistry is not, therefore, in any sense, the study of science. Such practice may depend upon certain scientific knowledge, but it is not of itself science." DR. W. C. BARRETT, Buffalo, N. Y.

